# A Comparative Evaluation of the Efficacy and Safety Profile of Cariprazine and Olanzapine in Patients With Schizophrenia in a Tertiary Care Hospital

**DOI:** 10.7759/cureus.91716

**Published:** 2025-09-06

**Authors:** Satyajyoti Tiwari, Brijesh Saran, Sivanesan Dhandayuthapani, Vivek Tejvir Yadav, Jyoti Batra, Saborni Dey

**Affiliations:** 1 Pharmacology, Santosh Medical College and Hospital, Ghaziabad, IND; 2 Psychiatry, Santosh Medical College and Hospital, Ghaziabad, IND; 3 Clinical Research, Santosh Medical College and Hospital, Ghaziabad, IND; 4 Biochemistry, Santosh Medical College and Hospital, Ghaziabad, IND

**Keywords:** antipsychotic efficacy, cariprazine, negative symptoms, olanzapine, schizophrenia

## Abstract

Background

Schizophrenia is a chronic psychiatric disorder requiring long-term antipsychotic treatment. Cariprazine, a newer atypical antipsychotic with D2/D3 partial agonist activity, has shown promise in managing both positive and negative symptoms. This study aimed to compare the efficacy and safety of cariprazine with olanzapine in patients with schizophrenia in a tertiary care setting in India.

Methods

A prospective comparative study was conducted among 60 patients diagnosed with schizophrenia, allocated in equal numbers to cariprazine and olanzapine groups. Baseline and six-week assessments were performed using the Brief Psychiatric Rating Scale (BPRS), Scale for the Assessment of Positive Symptoms (SAPS), Scale for the Assessment of Negative Symptoms (SANS), and the Udvalg for Kliniske Undersøgelser (UKU) Side Effect Rating Scale. Statistical analyses included t-tests, ANOVA, and chi-square tests, with significance set at p<0.05.

Results

Both groups showed significant improvements from baseline on BPRS, SAPS, and SANS scores (p<0.001). At six weeks, cariprazine was more effective in reducing negative symptoms (SANS: 39.90 ± 19.25) compared to olanzapine (SANS: 55.37 ± 16.22; p=0.005). Olanzapine led to a greater reduction in positive symptoms (SAPS: 38.13 ± 18.45 vs. 45.80 ± 20.33; p=0.008). UKU scores indicated minimal adverse effects in both groups, with cariprazine showing a slightly more favorable tolerability profile (2.97 ± 3.42 vs. 3.07 ± 3.63; p<0.001). Age significantly influenced BPRS outcomes, while gender, marital status, and socioeconomic status had no significant effect.

Conclusion

Both cariprazine and olanzapine demonstrated comparable overall efficacy. Cariprazine showed an advantage in improving negative symptoms and exhibited a more favorable side effect profile. Given the small sample size, short follow-up period, and single-center setting, these findings should be interpreted with caution. Larger, long-term trials are needed to confirm the results.

## Introduction

Schizophrenia is a chronic, debilitating psychiatric disorder that significantly affects thought, perception, emotion, and behavior. It typically manifests in early adulthood and leads to profound impairments in social and occupational functioning. Globally, the lifetime prevalence of schizophrenia is approximately 1%, with regional variation ranging from 1.2% in Canada and 0.6-1.9% in the United States to a lower rate of 0.42% reported in India [1,2]. Despite being well characterized, schizophrenia remains a major therapeutic challenge, particularly in low- and middle-income countries where access to individualized and tolerable treatment is often limited.

Pharmacological management primarily involves antipsychotic medications. First-generation (typical) antipsychotics such as haloperidol act mainly on D2 dopamine receptors but are frequently associated with extrapyramidal side effects. Second-generation (atypical) antipsychotics like olanzapine and risperidone act on a broader range of receptors, including serotonergic sites, and are generally preferred due to their better efficacy against negative symptoms (such as diminished motivation, blunted affect, and social withdrawal) and cognitive impairments [3]. Nonetheless, their long-term use is constrained by metabolic adverse effects and risks of treatment discontinuation due to relapse or poor tolerability [4,5].

Cariprazine is a newer atypical antipsychotic with partial agonist activity at D2 and D3 receptors, with stronger binding affinity for D3. This pharmacological profile distinguishes it from many other atypical antipsychotics and is thought to contribute to its greater efficacy in alleviating negative symptoms, which are often resistant to conventional treatment, while also maintaining activity against positive symptoms (e.g., hallucinations, delusions, and thought disorder) [6]. Olanzapine, on the other hand, is well recognized for its robust effect on positive symptoms but carries a significant burden of metabolic adverse effects, including weight gain, dyslipidemia, and insulin resistance [7,8].

These differences are particularly important in the Indian context, where pharmacogenetic variations (e.g., in drug metabolism genes such as CYP450 isoenzymes) and cultural factors (such as dietary patterns, treatment-seeking behaviors, and family support systems) may affect treatment response and tolerability [9,10]. Although studies in India have underscored olanzapine’s metabolic risks and limited cost-effectiveness, direct comparative data on cariprazine and olanzapine in real-world clinical practice remain scarce [11].

This study therefore aimed to evaluate the comparative efficacy and safety of cariprazine and olanzapine in schizophrenia within a tertiary care setting in North India. Specifically, it compared clinical outcomes and tolerability between the two agents, with the working hypothesis that no significant difference would be observed in efficacy or safety under similar treatment conditions.

## Materials and methods

Study design and setting

This was a prospective comparative study conducted over a period of one year (May 2024 to May 2025) in the Department of Pharmacology, in collaboration with the Department of Psychiatry, at Santosh Medical College and Hospital, Ghaziabad, India. The primary objective was to compare the clinical efficacy and safety of cariprazine and olanzapine in adult patients with schizophrenia. Ethical clearance was obtained from the Institutional Ethics Committee (Ref. No. SU/R/2024/1350(125), dated 17 May 2024). All procedures were performed in accordance with the Declaration of Helsinki and institutional guidelines.

Study participants

The study included adult patients aged 18-55 years diagnosed with schizophrenia based on the International Classification of Diseases (ICD)-11 diagnostic criteria, verified through structured clinical evaluation by consultant psychiatrists in the outpatient department (OPD). Both newly diagnosed patients and previously diagnosed individuals presenting with acute exacerbation of symptoms were eligible, provided they and their caregivers gave written informed consent. Exclusion criteria included patients with known hypersensitivity to either olanzapine or cariprazine, requirement of alternative psychiatric interventions (e.g., electroconvulsive therapy or depot injections), treatment-resistant schizophrenia, severe medical or neurological comorbidities, pregnancy or lactation, or concurrent treatment with drugs interacting with CYP3A4/2D6. Screening involved detailed history, physical examination, and medication review.

Sample size

Based on previous literature (Praveena et al.), assuming a medium effect size (Cohen’s d = 0.6), 80% power, and a 95% confidence level, the required sample size was 24 patients per group. To account for potential attrition, 30 patients were recruited per group, giving a total sample size of 60 participants [11].

Treatment allocation

Eligible patients were randomized into two groups in a 1:1 ratio using a computer-generated randomization sequence. Allocation concealment was ensured using sealed opaque envelopes. The olanzapine group received oral therapy starting at 10 mg/day, titrated up to a maximum of 20 mg/day depending on response and safety. The cariprazine group started at 1.5 mg/day, with dose adjustments permitted up to 6 mg/day based on clinical response. Dose titration was performed at weekly intervals. All participants continued routine psychiatric follow-up and psychoeducation throughout the study. Owing to the open-label design, blinding of patients and assessors was not carried out.

Data collection and outcome measures

Data were recorded on pretested case record forms, which included demographic and clinical details, and later entered into a secured Excel database (Microsoft, Redmond, WA, USA). Each participant was assigned a unique study ID to maintain confidentiality. Data accuracy was verified by random cross-checks against source records. Missing data were minimized and, where present, resolved using original clinical files.

Clinical efficacy and safety were assessed at baseline and after six weeks using the following validated instruments: the Brief Psychiatric Rating Scale (BPRS), used to assess the overall severity of psychiatric symptoms (18 items; score range 18-126; lower scores indicate improvement) [12]; the Scale for the Assessment of Positive Symptoms (SAPS), which has 34 items rated from 0-5 assessing delusions, hallucinations, and disorganized thinking [13]; the Scale for the Assessment of Negative Symptoms (SANS), which includes 25 items rated from 0-5 assessing flat affect, avolition, alogia, and anhedonia [14]; and the Udvalg for Kliniske Undersøgelser Side Effect Rating Scale (UKU-SERS), which consists of 48 items covering psychic, neurological, autonomic, and other side effects [15].

The primary endpoint was the mean change in BPRS total score from baseline to six weeks. Secondary endpoints included changes in SAPS, SANS, and the incidence and severity of side effects measured using UKU-SERS.

All assessments were performed by psychiatry residents trained in the standardized administration of the above scales, under supervision of senior faculty. Internal consistency of the scales was confirmed: BPRS (α = 0.830), SAPS (α = 0.804), and UKU-SERS (α = 0.801) showed good reliability, while SANS (α = 0.941) showed excellent internal consistency.

Statistical analysis

Data analysis was conducted using SPSS version 29 (IBM Corp., Armonk, NY, USA). Normality of all continuous variables was assessed using the Shapiro-Wilk test. Continuous variables that met the normality assumption were expressed as means ± standard deviations, while those that deviated from normality were summarized using median (interquartile range [IQR]). Categorical variables were summarized as frequencies and percentages. Between-group baseline comparisons were carried out using independent t-tests, Mann-Whitney U test for continuous variables and chi-square tests for categorical variables. Within-group changes (baseline to six weeks) were assessed using paired t-tests. Between-group differences in treatment outcomes were analyzed using independent t-tests. Additionally, ANOVA/ANCOVA models were applied to explore associations between demographic variables (age, gender, socio-economic status) and treatment outcomes. No corrections were applied for multiple comparisons, and findings are interpreted with awareness of potential Type I error.

Ethical considerations

All participants and caregivers received detailed information about the study and provided written informed consent. Patients retained the right to withdraw at any point without prejudice to their ongoing clinical care. Adverse events were systematically monitored and managed as per institutional safety protocols.

## Results

A total of 60 patients diagnosed with schizophrenia were enrolled and randomized equally between the two treatment groups: cariprazine (n = 30) and olanzapine (n = 30). At baseline, the cariprazine (n = 30) and olanzapine (n = 30) groups were comparable across key demographic and anthropometric variables. The mean age, gender distribution, and residence status showed no statistically significant differences between groups (p > 0.05). Both groups had a majority of urban dwellers and Hindu participants, with similar distributions in occupation, marital status, family type, dietary habits, and socioeconomic class. The average height, weight, and BMI were also similar across groups. No significant differences were observed in any of the baseline characteristics, indicating that the groups were well matched prior to treatment initiation (Table 1).

**Table 1:** Baseline Demographic and Clinical Characteristics of Participants in the Cariprazine and Olanzapine Groups (N = 60). BMI: Body Mass Index; p<0.05: p-value is considered significant

Variables	Caripraine (N=30)	Ofanzapine (N=30)	t-value/ χ2-value	p-value
	Frequency (%)/ Mean ± SD			
Age (years)	31.63 ± 8.03	29.87 ± 9.25	0.783	0.38
Gender				
Female	15 (50.0%)	14 (46.7%)	0.067	0.796
Male	15 (50.0%)	16 (53.3%)		
Residence				
Rural	5 (16.7%)	9 (30.0%)	1.491	0.222
Urban	25 (83.3%)	21 (70.0%)		
Occupation				
Farmer	2 (6.7%)	0 (0.0%)	4.877	0.431
Labourer	3 (10.0%)	3 (10.0%)		
Student	8 (26.7%)	9 (30.0%)		
Housewife	8 (26.7%)	8 (26.7%)		
Service	7 (23.3%)	4 (13.3%)		
Unemployed	2 (6.7%)	6 (20.0%)		
Religion				
Hindu	27 (90.0%)	27 (90.0%)	0.000	1.000
Muslim	3 (10.0%)	3 (10.0%)		
Marital Status				
Married	18 (60.0%)	16 (53.3%)	0.271	0.602
Unmarried	12 (40.0%)	14 (46.7%)		
Type of Family				
Nuclear	19 (63.3%)	16 (53.3%)	0.000	1.000
Joint	11 (36.7%)	14 (46.7%)		
Dietary Habit				
Non-vegetarian	10 (33.3%)	6 (20.0%)	1.364	0.243
Vegetarian	20 (66.7%)	24 (80.0%)		
Socioeconomic Status				
Lower Class	12 (40.0%)	10 (33.3%)	1.209	0.546
Middle Class	18 (60.0%)	19 (63.3%)		
Upper Class	0 (0.0%)	1 (3.3%)		
Height (m)	1.69 ± 0.09	1.65 ± 0.13	0.637	0.428
Weight (kg)	74.37 ± 9.76	70.80 ± 8.25	1.229	0.272
BMI (kg/m²)	25.71 ± 3.18	25.56 ± 2.56	1.863	0.178

The dosage distribution differed significantly between the cariprazine and olanzapine groups (χ² = 96.12, p < 0.001). In the cariprazine group (n = 30), the most frequently prescribed doses were 1.5 mg at bedtime (HS) in 17 patients (56.7%) and 1.5 mg twice a day (BD) in six patients (20.0%). Other dosages included 3.0 mg BD in four patients (13.3%), 3.0 mg HS in one patient (3.3%), 6.0 mg HS in one patient (3.3%), and 6.0 mg BD in one patient (3.3%). In contrast, among patients receiving olanzapine (n = 30), the majority were prescribed 10 mg BD in 19 patients (63.3%), followed by 10 mg HS in three patients (10.0%), 15 mg HS in two patients (6.7%), and 20 mg HS in two patients (6.7%). Other dosages included 5 mg BD in two patients (6.7%) and 2.5-5 mg HS in only two patients (6.7%) (Table 2).

**Table 2:** Comparison of Dosage Distribution Between Cariprazine and Olanzapine Groups (N = 60). BD: twice daily; HS: at bedtime; p<0.05: p-value is considered significant.

Dosage	Caripraine (N=30)	Ofanzapine (N=30)	χ2-value	p-value
	Frequency (%)			
1.5 mg HS	17 (56.7%)	0 (0.0%)	96.12	<0.001
1.5 mg BD	6 (20.0%)	0 (0.0%)		
2.5 mg HS	0 (0.0%)	1 (3.3%)		
2.5 mg BD	0 (0.0%)	0 (0.0%)		
3.0 mg HS	1 (3.3%)	0 (0.0%)		
3.0 mg BD	4 (13.3%)	0 (0.0%)		
5.0 mg HS	0 (0.0%)	1 (3.3%)		
5.0 mg BD	0 (0.0%)	2 (6.7%)		
6.0 mg HS	1 (3.3%)	0 (0.0%)		
6.0 mg BD	1 (3.3%)	0 (0.0%)		
10.0 mg HS	0 (0.0%)	3 (10.0%)		
10.0 mg BD	0 (0.0%)	19 (63.3%)		
15.0 mg HS	0 (0.0%)	2 (6.7%)		
15.0 mg BD	0 (0.0%)	0 (0.0%)		
20.0 mg HS	0 (0.0%)	2 (6.7%)		

At baseline, there were no statistically significant differences between the cariprazine and olanzapine groups in BPRS, SAPS, SANS, or UKU-SERS scores (p > 0.75 for all). After six weeks of treatment, significant differences emerged: the cariprazine group showed lower mean scores for SANS (39.90 ± 19.25 vs. 55.37 ± 16.22, p = 0.005) and higher BPRS (51.00 ± 9.44 vs. 46.70 ± 11.94, p = 0.041) and SAPS scores (45.80 ± 20.33 vs. 38.13 ± 18.45, p = 0.008), indicating greater impact on negative symptoms. Side effect severity assessed by UKU-SERS was comparable between groups but remained low overall (Table 3).

**Table 3:** Comparison of Mean Symptom and Side Effect Scores Between Cariprazine and Olanzapine Groups at Baseline and Six Weeks. *Independent T test; BPRS: Brief Psychiatric Rating Scale; SAPS: Scale for Assessment of Positive Symptoms; SANS: Scale for Assessment of Negative Symptoms; UKU-SERS: Side Effect Rating Scale; p<0.05: p-value is considered significant; † Mann–Whitney U test applied due to non-normal distribution (confirmed by Shapiro–Wilk test, p < 0.001).

Scale	Caripraine (N=30)	Ofanzapine (N=30)	t-value*	p-value
	Mean ± SD			
Baseline				
BPRS	57.30 ± 6.30	56.83 ± 6.15	0.287	0.775
SAPS	58.80 ± 12.85	58.23 ± 12.63	0.18	0.858
SANS	62.63 ± 12.70	62.33 ± 12.79	0.091	0.928
UKU-SERS	-	-	–	–
At 6 weeks				
BPRS	51.00 ± 9.44	46.70 ± 11.94	4.395	0.041
SAPS	45.80 ± 20.33	38.13 ± 18.45	6.636	0.008
SANS	39.90 ± 19.25	55.37 ± 16.22	6.977	0.005
UKU-SERS†	2 (1-5)	3 (1-6)	10.066	<0.001

Both treatment groups demonstrated statistically significant improvements from baseline to six weeks in BPRS and SAPS scores (p < 0.001), indicating a reduction in positive psychiatric symptoms. Cariprazine showed a greater decline in negative symptom scores (SANS) compared to olanzapine (t = 7.344 vs. 2.575; p < 0.001). UKU-SERS scores significantly increased in both groups over time (p < 0.001), reflecting emerging side effects, although baseline values were zero. Overall, both medications were effective, with cariprazine showing more pronounced improvement in negative symptoms (Table 4).

**Table 4:** Within-Group Comparison of Symptom Severity and Side Effects from Baseline to Six Weeks in Cariprazine and Olanzapine Treatment Groups. **Paired T test; BPRS: Brief Psychiatric Rating Scale; SAPS: Scale for Assessment of Positive Symptoms; SANS: Scale for Assessment of Negative Symptoms; UKU-SERS: Side Effect Rating Scale, p<0.05: p-value is considered significant.

Scale	Baseline	6 weeks	t-value**	p-value
	Mean ± SD			
Cariparine (N=30)				
BPRS	57.30 ± 6.30	51.00 ± 9.44	4.144	<0.001
SAPS	58.80 ± 12.85	45.80 ± 20.33	3.998	<0.001
SANS	62.63 ± 12.70	39.90 ± 19.25	7.344	<0.001
UKU-SERS	0.00 ± 0.00	2.97 ± 3.42	4.757	<0.001
Ofanzapine (N=30)				
BPRS	56.83 ± 6.15	46.70 ± 11.94	5.365	<0.001
SAPS	58.23 ± 12.63	38.13 ± 18.45	6.739	<0.001
SANS	62.33 ± 12.79	55.37 ± 16.22	2.575	<0.001
UKU-SERS	0.00 ± 0.00	3.07 ± 3.63	-4.632	<0.001

ANOVA results indicated a statistically significant difference in BPRS scores across age groups (F = 4.620, p = 0.006), with older patients (>45 years) showing higher mean scores, suggesting potentially less symptom improvement. No significant differences were found for SAPS, SANS, or UKU-SERS across age categories. Similarly, gender and socioeconomic status did not significantly influence any treatment outcome measures (all p > 0.05), although males had marginally higher BPRS scores than females (p = 0.065). Overall, age emerged as the only demographic factor significantly associated with treatment response (Table 5).

**Table 5:** Influence of Demographic Variables on Treatment Outcomes by ANOVA (with Comparison Groups). * Given the multiple comparisons made across demographic variables and treatment outcome scales using ANOVA, the risk of Type I error was addressed by applying the Bonferroni correction. The adjusted significance threshold was calculated by dividing the conventional alpha (0.05) by the number of comparisons made. For 12 ANOVA tests (3 variables × 4 outcome scales), the corrected significance threshold was p < 0.0042. Therefore, only results meeting this threshold were interpreted as statistically significant; BPRS: Brief Psychiatric Rating Scale; SAPS: Scale for Assessment of Positive Symptoms; SANS: Scale for Assessment of Negative Symptoms; UKU-SERS: Side Effect Rating Scale.

Demographic Variable	Comparison Groups	Scale	F-value	p-value
Gender	Male vs. Female	BPRS	3.539	0.065
		SAPS	0.729	0.397
		SANS	0.221	0.645
		UKU-SERS	1.503	0.225
Age Group (in years)	18–30, 31–45, >45	BPRS	4.621	0.006*
		SAPS	0.692	0.561
		SANS	0.763	0.524
		UKU-SERS	0.656	0.582
Marital Status	Married vs. Unmarried	BPRS	0.069	0.793
		SAPS	0.070	0.792
		SANS	0.498	0.483
		UKU-SERS	0.013	0.908
Socioeconomic Status	Lower, Middle, Upper class	BPRS	0.163	0.852
		SAPS	0.255	0.823
		SANS	1.514	0.238
		UKU-SERS	0.371	0.691

## Discussion

This study aimed to compare the efficacy and safety of cariprazine and olanzapine in the treatment of patients with schizophrenia at a tertiary care hospital in New Delhi, India. Given the unique pharmacological profiles of these drugs, particularly cariprazine's partial agonism at D2/D3 receptors and olanzapine's broader dopaminergic and serotonergic antagonism, our hypothesis proposed no significant differences between them in terms of outcomes [4,5]. However, the results demonstrate meaningful contrasts, particularly in symptom domain responses and dosing patterns.

Both cariprazine and olanzapine groups showed statistically significant reductions in BPRS, SAPS, and SANS scores from baseline to week six (all paired t-tests p < 0.001). These findings are consistent with previous studies by Li et al. and Rancans et al., which also reported notable within-group improvements in psychotic and negative symptom domains over six weeks of treatment [16,17]. This supports their short-term therapeutic effectiveness in schizophrenia. Notably, cariprazine achieved a greater reduction in SANS scores compared to olanzapine (39.90 ± 19.25 vs. 55.37 ± 16.22; p = 0.005). This difference highlights cariprazine's potential superiority in alleviating negative symptoms, an area where many antipsychotics fall short. Its mechanism of partial agonism at D₃ receptors may underlie this efficacy, aligning with preclinical data suggesting D₃ modulation enhances motivation and cognition [18]. Two pivotal trials by Németh et al. and Nasrallah et al. similarly reported that cariprazine significantly improved negative symptoms compared to risperidone, with effect sizes of 0.44-0.62, reinforcing our findings [18,19].

Our findings align with a case study Vrublevska et al., where after initiating cariprazine, which was combined with olanzapine, improvement in the patient's positive symptoms, negative symptoms, and overall functioning was detected [20]. Similarly, a study by Marder et al. confirmed cariprazine’s superiority in negative symptom domains across multiple Phase III trials, reinforcing our study’s results [21].

Cariprazine and olanzapine both reduced positive symptoms as reflected by BPRS and SAPS scores (p < 0.001). Between-group comparisons indicated that olanzapine may offer a slightly larger reduction in positive symptoms (t = 6.636 vs. 3.998 for SAPS); however, only cariprazine showed a statistically greater negative symptom improvement. This aligns with reviews by Batinic et al., showing that while typical antipsychotics (including olanzapine) are effective against positive symptoms, they often fail to adequately impact negative symptomatology. Our study thus supports cariprazine’s value in addressing these core deficits [22].

Regarding safety, both groups started with a UKU-SERS score of zero and showed a statistically significant increase by week six (p < 0.001). Although olanzapine produced a slightly higher mean increase (3.07) compared with cariprazine (2.97), this numerical difference is clinically negligible since both values fall within a low-to-moderate adverse effect range, suggesting that side effect burden was generally mild and manageable in both groups. While both drugs were tolerated, olanzapine’s high affinity for histaminergic and muscarinic receptors leading to its known metabolic and sedative side effect profile could explain its marginally higher UKU scores, consistent with findings by Men et al., demonstrating greater weight gain and dyslipidemia in olanzapine-treated patients compared to D₃-preferring agents [23]. Cariprazine’s lower histamine affinity likely contributes to its weaker sedative and metabolic footprint [24]. A systematic review by Pillinger et al. showed fewer metabolic disturbances with cariprazine compared to olanzapine over 12 months, supporting the tolerability trends observed in our short-term trial [25].

Cariprazine was mainly prescribed in the low-dose range of 1.5-3 mg/day (76.7%), while olanzapine was usually given at higher doses of 10-20 mg/day (90.0%). This dose pattern, confirmed statistically (χ² = 96.12; p < 0.001), reflects routine clinical practice where olanzapine requires higher absolute doses to achieve efficacy, whereas cariprazine is effective at lower doses. Such differences are important for real-world prescribing, as they may explain variations in side effect burden and highlight the practical advantage of cariprazine in patients requiring better tolerability. While our study did not include serum level measurements, pharmacokinetic variability, especially in Indian populations, has been reported in a clozapine case study by Charitha et al., though specific data on cariprazine is limited [26]. The reliance on lower effective doses for cariprazine 1.5-4.5 mg/day, as seen here and in may reduce the risk of adverse events without compromising efficacy, particularly in the negative symptom domain [27].

Demographic variables were largely non-influential in treatment response, except for age. Older patients (>45 years) had higher BPRS scores at follow-up (F = 4.62; p = 0.006), suggesting slower or less robust symptom resolution. This finding aligns with a study by Bajraktarov et al., who reported diminished response rates in older populations, possibly due to age-related pharmacokinetic alterations such as decreased hepatic metabolism or higher illness chronicity [28]. Neither gender, marital status, nor socioeconomic status showed significant effects, consistent with prior review by Periclou et al., which found that baseline symptom severity and duration of untreated psychosis were more predictive of outcomes than socio-demographics [29].

All assessment tools used in the study demonstrated strong internal reliability, with Cronbach’s α values ranging from 0.80 (UKU-SERS) to 0.94 (SANS). In particular, the excellent alpha for SANS (0.94) indicates highly consistent negative symptom assessment. These metrics validate confidence in the study results and align with reported psychometric performance in global and Indian populations [13-15].

Our study offers critical insight into real-world prescribing patterns in an Indian tertiary-care setting, reinforcing cariprazine’s utility in treating negative symptoms - a persistent treatment gap in schizophrenia care.

Limitations

Despite its strengths, the study’s six-week follow-up is short for capturing metabolic or cognitive outcomes. The small sample size (N = 60) and single-center design limit generalizability. Furthermore, side effect assessments focused on short-term adverse events; long-term metabolic monitoring is necessary. Future studies should include multicenter randomized controlled trials, extended durations (six or more months), weight/BMI monitoring, and cognitive endpoints. Incorporation of fixed placebo controls would further substantiate comparative efficacy evidence.

## Conclusions

This study provides comparative insights into the efficacy and safety profiles of cariprazine and olanzapine in the short-term management of schizophrenia. Both antipsychotics led to significant improvements in positive and negative symptoms; however, cariprazine showed greater efficacy in alleviating negative symptoms, while olanzapine was more effective in reducing positive symptoms. Cariprazine was also associated with a more favorable side-effect profile and required lower doses, suggesting its potential advantage in long-term tolerability. Although differences in treatment outcomes by gender, marital status, and socioeconomic status were not statistically significant, age showed a notable influence on symptom severity. These findings underscore the need for individualized, symptom-targeted pharmacotherapy in schizophrenia. The results also reflect the importance of considering regional and demographic variations in treatment response. Future multicentric, longer-duration studies with larger sample sizes are essential to confirm these findings and optimize antipsychotic strategies in diverse patient populations.
